# Integrating Quality Improvement: A Qualitative Study of Leadership Approaches in Healthcare Services in Norwegian Municipalities

**DOI:** 10.1177/11786329251403887

**Published:** 2025-12-23

**Authors:** Ingvild Røe, Maren Kristine Raknes Sogstad, Hilda Bø Lyng

**Affiliations:** 1 Department of Health Sciences in Gjøvik, Faculty of Medicine and Health Sciences, Centre for Care Research, NTNU – Norwegian University of Science and Technology, Trondheim, Norway; 2 Faculty of Health Sciences, Centre for Resilience in Healthcare SHARE, University of Stavanger, Norway

**Keywords:** quality improvement, Norway, healthcare services, organizational structures, delegation, bureaucracy, autonomous units, vertical, horizontal, mediating approaches

## Abstract

**Background/Objective:**

Healthcare services must provide high-quality patient care and continuously work to improve the quality of the organization. Continuous quality improvement (QI) involves systematic and ongoing enhancements of individual treatments as well as large organizational structures. To facilitate QI, suitable organizational structures are required. This study aims to describe and explore how administrative leaders organize for continuous QI in a Norwegian municipal setting.

**Methods:**

This qualitative study examines how administrative leaders organize for QI in Norwegian municipalities by conducting semi-structured interviews with leaders and quality advisors (N = 19) in three Norwegian municipalities. The data were analyzed inductively by reflexive thematic text analysis and the software NVIVO 14.

**Results:**

The municipalities used three main approaches to organize QI, aimed at integrating QI into their governing structures. The vertical approach aligned QI formalities with hierarchical structures. The horizontal approach created tailored structures for operationalizing and implementing QI. In the mediating approach, leaders actively and continuously worked vertically, horizontally, and across levels and units to reconcile differences, cooperate, communicate, and monitor activities, securing trust and commitment toward QI. Their responsibilities were extensive, supported by delegating responsibility to lower-level leaders and quality advisors, the latter with key roles in QI. QI functioned as a planned activity and continuous process. Trust and commitment were essential across approaches. Leaders’ continuous mediating activities helped address the tensions between autonomy and commitment, specialized or integrated assistance, and balancing change, trust and control. These were critical to QI’s success.

**Conclusion:**

The different approaches to organizing QI facilitated the implementation of QI in various situations and alleviated tensions regarding the autonomous units’ commitment to decisions and the integration or specialization of QI. The various approaches to QI, integrated with different bureaucratic models, created complex processes of layering new elements into existing structural forms, thereby modernizing municipal structures.

## Introduction

Good quality healthcare services should be safe, efficient, effective, equitable, accessible, patient-centered, and timely.^1,2^ In Norway, healthcare service organizations and professionals are legally obligated to provide good quality services and work continuously to improve quality throughout the organization.^2-4^ Quality improvement (QI) involves systematically planned and incremental improvements in all aspects, from individual treatments to large organizational structures. New treatments, more users with complex health problems, and an ageing population add to healthcare managers’ incentive to prioritize QI.^5-7^

To facilitate QI, public healthcare services must have flexible and appropriate organizational structures.^5^ Organizational structure can broadly be defined as a framework enabling coordinated effort between individuals.^8^

The overarching theoretical framework informing the results of our inductive data analysis is drawn from organizational and political science. This entails theoretical contributions concerning the development, structuring, and governance of formal public organizations.^9,10^ How the organizational structure is connected to individual and organizational behavior, decision-making, and implementation, and the distribution and enactment of leadership and power, is also valuable.^9,11-13^

The rational, formal, hierarchical bureaucracy is often the reference when describing and exploring public organizations, and remains the preferred organizational form in all sectors, worldwide.^9,10,14,15^ However, bureaucracy has also been criticized for being rigid, expensive, inefficient, and slow in resolving social and organizational trends and political reforms.^10,14,16^ Tensions of bureaucracy, such as autonomy versus accountability, trust versus control, or resistance to change, still inspire research and debate.^9,14^ Various theoretical models and new forms of bureaucracy have been developed to meet the critique. New public management (NPM) introduced private enterprise principles, competition, and measurement, and reinforced the importance of the unit’s autonomy.^9,10,14,16^ Post-bureaucracy (PB) promoted cooperation and informal and flat network structures based on autonomy, trust, and commitment.^16-19^ With post-NPM and new public governance (NPG), the focus returned to cooperation, horizontal integration, and coordination, but also renewed the interest in centralized governance.^10,16,20^ Hybrid models attempting to integrate conflicting structures within the same organization have also been promoted.^21^ The different models have had varied practical influences on public organizations within healthcare. In Scandinavia, traditional bureaucracy, NPM, and NPG have had a notable influence.^9,10,14,22^

Research also elucidated how QI should be structured to be successful. The foundations for such studies can be found in improvement research and implementation research.^13,23-26^ Organizational structures influence leadership, individual capability and relationships, organizational culture, capacity and capability, infrastructure, and readiness for change.^26-28^ Organizational structure has often been treated as “context” and, therefore, not analyzed independently.^12,29^ It is acknowledged that contextual factors are essential for QI, and by studying how leadership is conducted within QI, more knowledge can also be gained about context and organizational structure. Research on context as an independent factor has regained interest in organizational science.^13,30^

QI’s various aspects have been explored scientifically. Some studies have examined individual treatments, processes, experiences, and outcomes.^31,32^ Others evaluate different QI models.^24,31^ It is also found that resources, leadership, commitment, communication, resilience, and flexibility have a positive impact.^12,33,34^ QI can improve structure, processes, and treatments. It adapts to context, challenges barriers, and overcomes resistance to change.^5,24,31,35^ However, despite positive practical experiences from QI, long-term effects have been more varied.^23-25^ Research efforts on the organization of QI have often concentrated on hospital care and professional/department leaders. We aim to describe and explore how administrative leaders organize for continuous QI in a Norwegian municipal setting, specifically at the top administrative levels.

## Methods

### Setting: Norwegian Local Government and Healthcare Services

Norwegian municipalities are autonomous local government legal entities with popularly elected councils.^36^ They make decisions but also concede to the national parliament’s resolutions. As “generalist” organizations, they are legally obligated to provide the same types and levels of services to everyone in the municipality.^37,38^ According to local autonomy grants, the administrative top leader organizes municipality and health services per local needs.^37^

**Figure 1.**
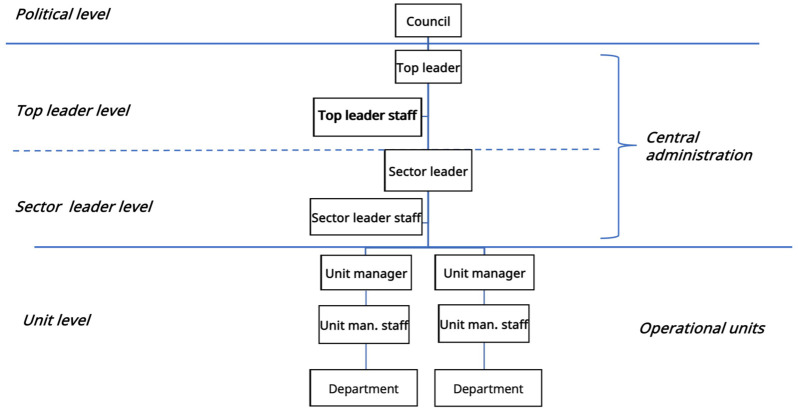
Municipality: Organization and decision line.

Norwegian municipalities are typically hierarchically structured and divided into a political level (council and mayor), central administration with a top level (top leader/chief municipal executive), and sector level (sector leader/municipal director); the unit level entailed autonomous operational units (unit manager/unit; Figure 1).^8,36,38,39^ In our study, administrative leaders on each level had a staff of subordinate functions, comprising advisors of relevant competence (quality advisor, staff).

Municipalities are governed by a system of 12- and 4-year master municipal plans and budgets outlining the council’s vision, strategic goals, and actions for municipal service provision, operationalized in economic plans and annual budgets allocating resources to different sectors/services. Operational units are autonomous and responsible for their budget, personnel, and quality of service. The management is framed by formal decision-making and delegation structures anchored in the municipal plan.^36,37^ Delegation means that the leader formally assigns responsibility for a task to someone with the right competence at a lower level, but remains accountable for the result. The administration monitors and reports progress on the goals and budget to the council, and conducts internal control and audits to ensure that legislation and decisions are followed.^36^

We explore QI in local municipal healthcare services. Healthcare services in Norway are organized and managed on different yet interdependent levels and framed by national laws and regulations.^2,3,40^ State-owned hospital trusts provide specialist health treatment. Municipalities offer healthcare services to people of all ages, primarily by securing available general practitioners, operating home-care services, and providing nursing homes.

### Participant Selection and Description

To gain new in-depth knowledge on how leaders at top administrative levels organize QI work, we conducted a qualitative interview study. We were not aiming for generalizations. Still, to ensure a broad selection of participants, internal similarities and differences, and a number aligned with information power recommendations, we strategically recruited one municipality from each of Norway’s central, eastern, and inland regions, a total of three municipalities, from same municipality group in Statistics Norway; medium sized (30 000-45 000 inhabitants) with similar economic frames. Information power is not calculated, but the number of interviewees (N = 19) was set following recommendations in the literature (15-20 interviewees to reach 95% coverage).^41^ Potential participants were top administrative leaders and quality advisors in central administration and operational units. Frontline staff, end users, and politicians were omitted because the study’s aim was limited to top-level administration.

Once recruited, our contacts in the municipalities were asked to set up interview appointments with participants in the decision line (Figure 1): the top leader, sector leader, two healthcare service unit managers, one staff quality advisor from each organizational level. Our contact chose all participants. We interviewed 12 administrative leaders and seven quality advisors (N = 19). To our knowledge, no one refused to participate. The number of advisors varied due to different organizational structures and vacancies in positions we did not know beforehand.

Table 1 presents the participants divided by municipality and position; 15 were health professionals, and the rest were educated in political, economic, and educational science. Most leaders had formal management education, most advisors had completed non-credit QI courses, and some had formal QI education. The majority had been employed for over 3 years in their current position and had lots of experience in the sector. Further specification of the participants is sensitive information.

**Table 1.** Participants Divided by Position and Municipality.

Participant position	Municipality 1	Municipality 2	Municipality 3	Total
Chief municipal executive/top leader	1	1	1	3
Top-level staff		1		1
Municipal director/sector leader	1	1	1	3
Sector-level staff	1	2	1	4
Unit manager	2	2	2	6
Unit-level staff	1		1	2
Participants’ total numbers	6	7	6	19

Table 2 describes the roles and titles at different organizational levels of the administration and the name/abbreviation used in the text and quotations.

**Table 2.** Description of Participant Titles/Roles and Name/Abbreviation Used in Text/Quotations.

Organizational level	Title/role	Description	Name/abbreviation
Central administration	Municipality	The administrative leadership of the municipality, including central administration and operational units	Municipality
Top-level	Chief municipal executive	The highest-ranking administrative leader of the municipality, answers to the council	Top leader/CME
Sector level	Municipal director	The highest-ranking administrative leader of the healthcare sector; answers to the top leader	Sector leader/MD
Operational or unit-level	Unit manager	Leader of the operational unit delivering healthcare services; autonomously responsible for personnel, economy, and service quality	Unit manager/UM
	Operational unit	operational unit delivering healthcare services	Unit
All levels	Quality advisor	Personnel in staff roles; responsible for QI in central administration and/or units	Quality advisor/QA
	Quality lead	Advisor in central administration, responsible for QI in the municipality, advisor to all units	Quality lead/QAC
	Staff	Staff for leaders at different levels	Staff

### Methods for Data Collection and Analysis

Qualitative interviews were conducted to explore participants’ experiences, attitudes, meanings, and reflections on organizing QI.^42,43^ Interview data were collected from March 2 to May 22, 2023. Plans and documents describing municipalities’ QI organization and observations in a quality committee meeting in each municipality were also collected to triangulate the data. Following recommendations from NSD Sikt’s approval of the research protocol (Ref.nr. 923911), municipalities and participants were informed about the aim, the researcher’s position, the possibility of withdrawing consent, providing written consent, and anonymizing, in writing beforehand, and presented at the start of each interview. The Consolidated criteria for reporting qualitative research (COREQ) checklist was followed (see form in Supplemental File).^44^ The semi-structured interviews had the following themes: QI structure, plans and processes, role in QI, prioritizing, challenges, implementing, anchoring, commitment, legal obligations, communication, cooperation (see interview guide in Supplemental File).

QI is defined as the participants’ understanding of the term, not any specific QI model. The main author, an MD, is experienced in qualitative studies, and the full research team has high expertise in this area. The main author conducted all interviews in Norwegian (average 50 minutes). The co-author participated in one interview. Interviews were taped and transcribed verbatim by the main author and a professional transcriber. Observations in meetings were documented during the meeting based on an observation guide (see guide in Supplemental File).

#### Reflexive Thematic Text Analysis

Data were analyzed inductively, following reflexive thematic text analysis principles by Brown & Clarke.^42,43^ Interviews were read, and initial codes were set close to the text by the main author. During the coding process, co-authors reviewed some interviews and codes from the analysis program NVivo 14 to be familiar with the material. After coding all interviews, the codes were sorted into possible main themes. In analytical workshops, all authors had many discussions of possible interpretations of codes and themes, and interviews were reread for missing or misinterpreted codes. This process was repeated several times in a reflexive search for integrations and refining themes, and several drafts were made before agreeing on a coherent representation of the material. There were no special disagreements, and all authors agreed on the final text. Lastly, the findings were translated into English.

Through the reflexive thematic text analysis, we identified the following themes and subthemes (Table 3). NVivo 14 was used for documentation and transparency.

**Table 3.** Main Themes and Subthemes.

Main theme	Subtheme
Organizational structures	Making QI an integrated part of the formal structures (vertical)
	Tailored structures QI implementation (horizontal)
Delegation	Commitment and ownership
	Monitoring and control
	Cooperation and communication
QI prerequisites	Trusting relationships
	QI as a process

## Results

Results are presented with a few quotes to illustrate the main themes. Table 4 in the Supplemental Material presents examples of how we analyzed text to code to theme and subthemes from each of the main themes.

### Main Theme 1: Organizational Structure

The first main theme concerns organizational structures for quality improvement (QI). Municipalities employed different strategies to establish comprehensive frameworks. A vertical approach aligned QI efforts with hierarchical decision-making processes, emphasizing strategic planning, prioritization, and governance (Figure 1). In contrast, the horizontal approach focused on developing tailored structures at the operational level, facilitating implementation and coordination across similar units.

#### Making QI an Integrated Part of the Formal Structures

The intention was to integrate QI into the entire municipal organization and make it a natural process. As such, QI had to be integrated across all vertical levels and focused on leader agreements and leader meetings. Strategy and operations were decided in leader meetings at every level. It would not be sufficient to discuss QI only at the superior level; it had to be integrated from top to bottom, and everybody had to get invested in the need for QI.

The first step toward this was utilizing the bureaucratic structure to integrate QI into the municipality’s governing system by including and operationalizing QI into municipal service provision and budget strategies. This top leader pointed out the importance of cooperation between leaders, staff, and personnel at all levels:

We are currently working to integrate this [QI-structure] so that top leaders can work closely with those engaged in QI. We expect leaders and quality advisors to work together in partnership (CME1).

The municipalities were at different stages. Some were still determining how and how often QI should be discussed in various arenas and forums. Still, all had started to establish some routines for handling QI issues and implement a comprehensive quality system. One quality advisor explained what this entailed:

We have a plan, although we are not quite there yet. Still, we have taken important initial steps – reallocating resources, establishing a new organizational flow, and adopting a fresh mindset. These changes have been positively received, and we are already beginning to see encouraging results (QA1).

We found that leaders felt ownership toward QI and wanted to contribute to positive attitudes. However, the leaders’ focus shifted with each organizational level. The municipality’s top leaders focused on structuring QI, making strategic decisions, implementing internal controls, supporting sector leaders, and collaborating with politicians. Sector leaders were delegated responsibility for healthcare and QI; therefore, they focused on developing service and QI strategies, monitored and responded to units, quality committees, and other QI structures, and contributed to the top leader team and the top leader’s cooperation with politicians. Unit managers ensured QI in their unit, solved daily QI questions, supported the sector leader, and cooperated with other units. All leaders required support, and should seek guidance from their quality advisors and other staff to manage their workload effectively.

QI initiatives were implemented both top-down and bottom-up within the structure. Typically, from the top, revision reports, deviations, or national guidelines prompted an initiative to mobilize the vertical structure. Simultaneously, experiences and suggestions from the bottom initiated action to raise an agenda at the top. This involved interactions between leadership levels. Top leaders anchored responsibility top-down, whereas subordinate leaders anchored their assignments bottom-up.

#### Tailored Structures for Implementing QI

The horizontal approach showed how tailored resources, structures, and arenas supported QI work at the organizational level. All municipalities had sector-level quality committees where mostly unit quality advisors, led by sector quality advisors, collaborated to operationalize and implement QI, and which supported the sense of shared responsibility for QI:

I believe the quality committee, with representation from all units, is positive, as it fosters collective ownership of quality improvement efforts (QA2).

Observations supported the notion that the quality committees were at different stages toward being key QI arenas for leadership support, cooperation, facilitating and monitoring actions along the decision line, and operationalizing QI issues between levels and across units. The committees did not possess the same formal status as the leader meetings and therefore needed to clarify major issues with the sector leader meeting.

**Figure 2.**
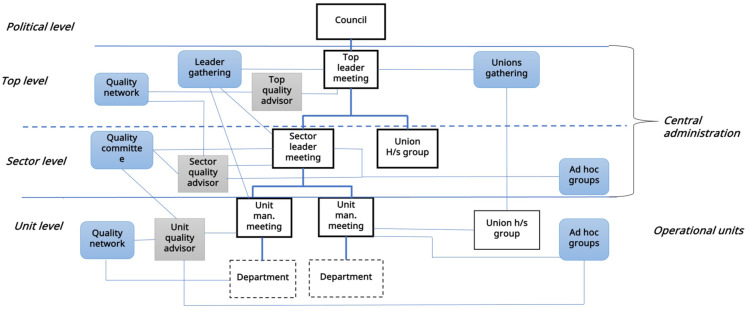
Quality improvement organization.

Municipalities also established networks for quality personnel to share experiences, for instance, on the use of the quality system. Additionally, ad hoc groups were formed to investigate specific issues related to planning new services or developing care pathways. Other ways to support leaders and units in QI were to simplify and standardize processes, develop templates, and unify routines. Figure 2 represents the QI organization described in this section, with formal and customized arenas.

Besides the involvement in tailored structures and standardizations, we found that the quality advisors were a key factor and essential resource in QI. The quality advisors provided crucial day-to-day support for the leaders, advised units, and participated in or led quality committees and networks. They planned, monitored, reported on QI activity, and often administered quality systems. However, the role had its limitations, especially the opportunity to make decisions:

Our role is somewhat floating—we lack dedicated resources and decision-making authority, yet we are expected to maintain clarity about our function and how we are utilized (QA3)

Owing to organizational differences and vacancies, the quality advisors were, in general, few (usually one per unit) and unevenly distributed between levels and municipalities. The need for help among units and the leader’s requirement for progress have prompted some municipalities to start building the competence and role of quality advisors as facilitators. This is achieved by educating them in relevant QI topics and adjusting their role to that of practical assistants. The unit managers highly appreciated this as it helped them and department managers who were otherwise pressed for time, and it was essential for speeding up the QI processes. However, quality advisors could experience a dilemma concerning how to conduct this assistance: Should they help and teach units become independent, thereby integrating QI as part of the unit’s work, or do the task for them now, leaving QI as a specialized task performed by dedicated quality advisors, but maybe hinder the integration of QI work in everyday tasks. Quality advisors described this as a difficult balance:

Maintaining the right balance is challenging; while units require support to implement changes, there’s an ongoing tension: should we keep holding their hand, providing guidance, or fostering their ownership and let them be independent next time? (QAC2).

Municipalities differed in how QI was coordinated. Two municipalities divided this function between advisors at different levels; one had a designated quality lead who was responsible for the municipal internal control system and assisted leaders in creating a comprehensive QI structure of unified routines, checklists, and procedures. Together with the top leader, sector leaders, and units, the quality lead had been crucial in developing a shared QI culture and aiding leaders to recognize that creating a QI structure was their responsibility.

### Main Theme 2: Delegation

Leaders at each level had numerous responsibilities and assignments related to QI (and other issues), and they required support in managing QI. This was provided by delegating responsibilities and assignments to lower-level leaders and quality advisors. Based on this distribution of responsibility and tasks regarding QI work, delegation was set as the second main theme.

#### Ownership and Commitment

Delegation, or assigning duties and tasks to others at lower levels while still being responsible, was considered an effective method to confirm ownership and commitment and clarify responsibility and accountability; if used as designated, it eased leaders’ workload. The leaders ensured that everybody understood the importance, took ownership, was committed, implemented QI, and recognized that it required teamwork. Maintaining this unity could be challenging; the units were different and required frequent attention in leader meetings and gatherings. The system of delegation and anchoring was on its way to being good:

The units are different, of course. . . There are differences in team cohesion and anchoring. However, from top leadership to sector leaders and unit managers, there appears to be strong alignment, constructive dialogue, and a shared commitment to quality (UM3).

Some leaders required additional support to understand delegations and sought confirmation or reinforcement from top leaders. Top leaders confirmed involvement and commitment by anchoring assignments with the lower-level leaders. This “double insurance” activity was time-consuming and could indicate that management or delegations needed clarification. This could potentially reveal management or delegation inadequacies:

Maybe it’s just how our system works, or how we do management, that anchoring decisions at the top appears necessary, even though we have a system characterized by broad delegation? (CME 1).

We found that in general, all leaders were committed to implementing resolutions from leader meetings, quality committees, networks, and ad-hoc groups. Nonetheless, delays, postponements, and non-compliance with decisions could occur. This could be owing to a lack of resources, prioritization, or appropriate competence, as well as incomplete and poor anchoring, loose commitment, and insufficient leadership. Sometimes, no apparent cause could be identified, leading to uncertainty among the leaders about how to respond. Resistance (hidden or open, specific or general) also could prevent compliance and progress:

We now practice a more collective form of leadership, with shared responsibility for the whole. . .But still, there’s resistance. People don’t always say it out loud, but it’s there - . . .whether the change is good or bad, the resistance is the same (MD1).

Even though we found movement towards a more unity-focused approach, the resistance from autonomous units was the most challenging to capture and resolve at present.

#### Monitoring and Control

Leaders often felt frustrated with the units’ lack of compliance. They implemented several actions to ensure that the units followed up on their responsibilities and tasks, delivering the desired results. One unit manager explained how tight they could need to be:

As leaders, we have to monitor closely. Sometimes we even have to say, ‘Today we’re doing this - who is doing it?’ or just do it ourselves. If we’re not close enough, things fall apart. You must be hands-on. And I have noticed that if we focus on other things, they stop doing it! Leaders must remain present and responsive to sustain momentum (UM2).

Actions ranged from reminding and repeating everything, appealing to the unit’s commitment in leader meetings, calling for status reports, formally delegating issues, and accepting responsibility in leader agreements. The leaders experienced that individually “leading the leaders” to ensure they did what they were supposed to, held the most promise.

We found that the central administration also conducted various monitoring and control activities to ensure that the sectors and operational units delivered results, maintained control over the budget, and that leaders had the appropriate authority. This was reported to the council through various documents, plans, and reports. They put significant capacity and effort into this. Economic status was reported regularly to the council. Results on the main municipal plan and budget were typically reported yearly. The annual report included QI; otherwise, municipalities differed in how often they reported QI. Sector leaders monitored units through self-evaluations or checklists, tracking both national and internal indicators.

#### Cooperation and Communication

To support QI, the leaders continuously worked and cooperated vertically, horizontally, and across levels and units to answer questions, make quick decisions, reinforce trust, monitor processes, secure units’ commitment, and reconcile strategic, administrative, and political issues. All this was accomplished through extensive communication and interaction in meetings, phone calls, and emails. Both “traditional” meetings, posters, and newsletters, and “new” digital platforms and systems were used for information and cooperation purposes. The numerous meetings also provided an opportunity to discuss and clarify issues, as well as communicate decisions.

We found that this continuous activity comprised an independent “mediating approach” for leaders in QI work, representing a significant part of how leaders contributed to QI. The mediating activity required close relations among leaders and individual capability to recognize when delegation was not feasible and rectify discrepancies. To avoid misunderstandings, leaders had to trust their own judgments and be quick to resolve them.

I can identify the decisions that need to be brought to the top leader’s attention, as we are very aligned and have a dialogue on this matter (MD 1).

Analysis showed that, due to time constraints, leaders were reluctant to bring their uncertainties to their superiors’ attention, and the opportunities for informal discussions with peer leaders for advice varied between municipalities.

Internal and external information and communication were essential leadership activities. Municipalities provided information and arranged digital Teams meetings to address personnel’s calls. However, it was not easy:

It is easy to think that once you’ve sent an email or mentioned something in a personnel meeting, the message is delivered. This contradicts fundamental aspects of human communication. You need to repeat and say things in a different way for it to stick. If a leader doesn’t get that, it becomes a barrier, I think (QA3).

Another arena fostering cooperation was the “tri-party collaboration” between the municipal administration, politicians, and workers’ unions. This is a core element in Norwegian governance; leaders emphasized integrating QI into these arenas.

### Theme 3: QI Prerequisites

Throughout our analysis, mutual trust appeared as the foundation for all the municipalities’ approaches to implementing QI. Another prerequisite was to recognize that QI comprises planned and continuous/incremental systematic processes, and that change takes time. These foundations for QI constitute the third main theme.

#### Trusting Relationships

The analysis showed that although the horizontal approach was more dependent on trusting relationships between otherwise autonomous units, it was also key in the vertical formal approach, ensuring commitment to the bureaucratic structure. Trust was crucial for everyone, both individually and embedded in the organization, and especially the leaders:

When leaders deflect responsibility or fail to understand the purpose of QI, don’t understand this themselves, or simply say ‘we were told to do this’ – they undermine trust in the process and weaken its legitimacy (CME2).

The freedom to act that follows from this mutual trust was highly valued, enabling leaders and staff to work for QI and invent new solutions in all parts of the services without close supervision or stringent control. Leaders had to trust that everyone brought different competencies and perspectives to the process and allowed personnel to work without constant monitoring.

Concurrently, this freedom was limited to situations where responsibilities were reconciled. The leaders felt they could do things they wanted, provided this was within their authority and budget. Their nearest administrative leader was not always involved in such actions. If they wanted to start a project involving multiple units or extra resources, they needed to involve and get approval from higher-level leaders.

#### QI Is a Process

Municipalities stressed the importance of systematically working on QI. This entailed establishing a plan, framework, or joint model for QI, and recognizing that QI is an iterative and time-consuming process that involves a gradual and ongoing cycle of improvement. One sector leader:

To meet our obligations, we established a top-level intra-sectoral group focused on internal control, so that’s in order. And we (sector level) are progressing from the sector to units gradually downwards. I think we’ve got pretty good ‘order in our house. But it’s ongoing – you’re never really done. (I: unfortunately?) Or maybe fortunately! (MD3).

They wanted to plan activities and simultaneously improve everyday issues, even if this was not part of the plan. It was also important to pause and appreciate what they had accomplished, fostering motivation to continue improving. Our findings confirmed that municipalities had defined strategies and set up systems to realize QI. The goal of getting this implemented and used by everybody remained to be solved.

Our main challenge lies in having the time, capacity, and authority to follow through on the goals we’ve set. The critical question remains: do we actually implement what we commit to? (QA2)

## Discussion

The municipalities employed different strategies to organize QI, seeking to integrate it with governing structures. The vertical approach aligned QI formalities with hierarchical structures, while the horizontal approach established tailored structures for implementation. The mediating approach combined both: leaders worked across all levels and units to reconcile differences, build cooperation, communicate, and monitor activities, fostering trust, commitment, and positive attitudes toward QI. Their responsibilities were extensive, supported by delegated tasks to lower-level leaders and quality advisors, the latter playing key roles in QI. QI functioned as a planned activity and continuous process. Trust and commitment were essential across approaches, while leaders’ mediating activities helped address tensions between autonomy and coordination, commitment and change, trust and control, and specialization versus integration. These were critical to QI’s success.

To better understand municipal QI organization, we connect these approaches with new research on public organizations, bureaucratic forms, and QI structuring.^13,23-26^ Traditional bureaucracy, with hierarchical structures, formal decision lines, and top-down delegation, has long been prevalent, though often criticized as rigid and inefficient.^8,9,14,16^ In response, new bureaucratic forms have sought to improve internal and external structure and increase efficiency, cooperation, and service quality.^10,14,16^

New Public Management (NPM)^14,39^ emerged to address inefficiency and rising costs, advocating private-sector principles, measurement, and competition to improve quality at lower cost. Power should be decentralized to autonomous, results-oriented units.^10,14,16^ Although NPM’s effectiveness is debated and its popularity has declined, its emphasis on autonomy and measurement persists in many Norwegian municipalities.^30,39,45^

Post-bureaucracy (PB) advocates for the empowerment of autonomous personnel and units that work together in flat structures, flexible cross-functional teams, and networks based on commitment, trust, informal relationships, and open communication.^9,17,18^ However, the PB’s preference for unity could complicate decision-making, especially if opinions differed.^14^ As a reaction to the fragmentation and decentralization of NPM, the ideas of PB reappeared with post-NPM and New Public Governance (post-NPM/NPG). These models still rely on cooperation and teamwork as organizational principles, but to enhance effective decision-making, reintroduced centralized management/governance.^16,45,46^ PB and post-NPM/NPG have not impacted the public sector as widely as NPM. Still, trusting, empowered, and committed personnel working in teams is part of the work in many public organizations.^22^

Our findings reflect elements from several organizational models. Municipalities’ organizational charts reveal a traditional bureaucratic hierarchy.^9,14^ The vertical approach, integrating QI into formal leadership structures, resembles this model and supports decision-making, funding, and setting mandates.^14,15^ Choosing to embed QI in governance rather than in a specialized unit aligns with both QI literature and Norwegian legislation.^4,23,24^

The horizontal approach tailored structures for implementation through quality committees and networks, consistent with PB and post-NPM/NPG preferences for informal cooperation based on trust, commitment, and communication.^9,18,46^ Yet, trust and commitment also underpinned vertical decision systems.^17,18^ Post-NPM/NPG’s emphasis on centralized governance reinforced both approaches, since committee suggestions required formal approval by leaders.^10,30^ These arrangements reflect structures described in successful QI studies.^23-25^

The mediating approach combined vertical and horizontal work: leaders anchored decisions, monitored progress, coordinated across levels, and cultivated trust and commitment. Depending on context, they drew on traditional bureaucracy, NPM, PB, and post-NPM/NPG. Literature emphasizes the need for close cooperation between administration, units, and professionals, ideally coordinated at a high organizational level.^23^ Municipalities met this by distributing responsibility among sector leaders, unit managers, quality advisors, and quality committees, or in one case, a quality lead.

Research advocates for flat structures because minimal management layers/levels between the top leader and units^17^ foster creativity and quick decisions, well-suited for QI.^9,14,17,23^ PB and post-NPM/NPG also favor such models. While municipalities did not adopt this overall organizing principle, they used team-oriented internal structures, ad hoc work groups, and a leader team to achieve similar effects.

Because QI is about change while organizations emphasize stability, tensions arose.^14,47^ Informal approaches challenged hierarchical decision-making, while vertical structures sometimes constrained creativity but also formalized innovative solutions. Within the mediating approach, leaders combined models to balance tensions and ensure effective QI. Ultimately, success depended on leaders’ ability to identify the appropriate approach and adapt principles from different models.

Committed leaders and trusting relationships were the foundation of QI work, shaping all approaches and bureaucratic forms. Trust can be vertical, horizontal, internal, and between units, and high and low levels of trust are mutually reinforcing.^48,49^ Commitment influenced attitudes: committed leaders engaged actively and succeeded in implementing QI.^49^ While participants often viewed trust as a personal quality, it was also embedded structurally – for example, council trust determined a top leader’s employment. Lack of trust, commitment, or cooperation made QI difficult.^9^ When trusted, personnel and leaders could work with relative autonomy, but doubts about intentions led to tighter control.

Operational services were organized in autonomous units, with managers who could approve or reject higher-level decisions. Horizontal and mediating approaches, and all the new bureaucratic forms, supported autonomy, but for different reasons.^11,16^ PB and post-NPM/NPG encouraged units to cooperate autonomously in trusting relationships.^17^ NPM regarded autonomous units as independent operators of service based on effectiveness.^16,39^ Despite tensions, autonomy remains one of NPM’s most enduring principles in Norwegian municipalities.

Leaders and quality advisors carried heavy workloads with limited resources, restricting their attention to QI. Delegation and simplification helped, while quality advisors provided daily support, but were few and time-constrained.^12,23^ Their role raised dilemmas: whether to integrate QI into routines through teaching or manage it directly as specialists.

Organizations failing in QI experience barriers such as hierarchical leaders with limited ownership, resistant personnel, scarce resources, and heavy workloads.^23,26,25,50^ Success depended on leadership, organizational culture, and motivation for change.^3,23,26,50,51^ Municipalities with strong quality cultures, sufficient resources, and committed leaders were more likely to succeed.^28,50,51^

Leaders addressed resistance through appeals, repeated messaging, monitoring, and formal reporting. Resistance was also shaped by vertical power, prior experiences and unit autonomy.^16^ To gain insight, implementation science offers useful perspectives for applying QI in practice.^52^ Leaders often relied most on personal follow-up, which was crucial in mediating the approach of informal dialogue and cooperation across levels and units.

Overall, municipalities can build bureaucratic models to implement QI. The vertical approach complemented traditional bureaucracy. The horizontal approach reflected PB and post-NPM/NPG’s emphasis on cooperation and trust, commitment, and positive attitudes. Yet bureaucratic forms could also hinder QI by reinforcing resistance to change. Although municipalities did not abandon their vertical governing structures, they layered new elements into existing structural forms, recreating municipalities as modern organizations.^10,16^

This study has both strengths and limitations. To our knowledge, this is among the first studies to describe and explore how administrative leaders organize for continuous QI in a Norwegian municipal setting. Municipalities vary in geography, population, and economy. Although all are legally required to provide and improve quality, their QI structures may differ. To gain in-depth insight, we chose a qualitative design in a few municipalities of similar size. This limits transferability, as our data cannot represent the views of the entire municipality. Also, frontline staff and end users were omitted because the study’s aim was limited to examining how top administrative leaders structure QI. The study design did not include follow-up or assessment of sustained improvements linked to specific QI models or interventions, nor did it incorporate comparisons with quality indicators, patient safety metrics, health-related outcomes, or other quantitative data. These limitations highlight the need for future research that accounts for variations in size, location, other stakeholders, or sustainable improvement from QI models. Investigating potential correlations between our study and measurable QI outcomes also represents a promising avenue for further research.

Information power is not calculated, which is a weakness, but the number of interviewees (N = 19) is carefully set according to literature recommendations, estimating 15 to 20 interviewees to achieve 95% coverage.^41^

The main author is employed as an advisor and secretary for the senior committee in one of the studied municipalities, which raises potential for bias. As a member of the sector leader staff, the author has insight into QI issues and familiarity with the leaders who were interviewed, having also participated in leader meetings. Although the author had no conflicts with participants, there was a risk that familiarity might limit disclosure of negative opinions. To mitigate this, the author did not select participants; interviews were conducted privately in the participants’ offices, and participants were fully informed about the study, the author’s dual role, and the emphasis on confidentiality. Interviews were inductive, with participants determining themes, while the guide served only as a prompt for reflection.

The topics addressed were not particularly sensitive or controversial, and all participants decided how much they wanted to share, based on the topics in the interview guide. No questions were refused, and all responses were handled discreetly in line with NSD Sikt’s recommendations.

Familiarity with the municipal context is a proven strength, particularly in the analysis and interpretation of data. The main author’s detailed knowledge of how municipalities are governed has enriched the study. At the same time, the risk of bias was actively monitored: co-authors reviewed all findings, discussed interpretations, and ensured that results from one municipality did not overshadow others. The results were overall consistent between all municipalities.

## Conclusion

Municipalities implemented QI using vertical, horizontal, and mediating approaches, aiming to integrate QI with the formal governing structure. These approaches for QI, combined with elements/structures from bureaucratic models (PB, NPM, and post-NPM/NPM), created complex processes of layering new elements into existing structural forms. This contributed to modernizing the municipal organization and allowed for a flexible and effective integration of QI. Trusting relations and committed personnel were QI pillars. To reduce workload, leaders utilized the system of delegation supported by quality advisors/facilitators, who were key QI resources. The different approaches to QI entailed some tensions/dilemmas. The autonomous units’ resistance and the balance of specialization and integration of QI could potentially delay or hinder QI progress.

This study highlights structures and tensions concerning how leaders navigate QI, which top leaders should address in their future efforts to integrate QI within formal governance. Further research is also needed to explore the proposed approaches and layering processes.

## Supplemental Material

sj-doc-1-his-10.1177_11786329251403887 – Supplemental material for Integrating Quality Improvement: A Qualitative Study of Leadership Approaches in Healthcare Services in Norwegian Municipalities Supplemental material, sj-doc-1-his-10.1177_11786329251403887 for Integrating Quality Improvement: A Qualitative Study of Leadership Approaches in Healthcare Services in Norwegian Municipalities by Ingvild Røe, Maren Kristine Raknes Sogstad and Hilda Bø Lyng in Health Services Insights

sj-docx-2-his-10.1177_11786329251403887 – Supplemental material for Integrating Quality Improvement: A Qualitative Study of Leadership Approaches in Healthcare Services in Norwegian Municipalities Supplemental material, sj-docx-2-his-10.1177_11786329251403887 for Integrating Quality Improvement: A Qualitative Study of Leadership Approaches in Healthcare Services in Norwegian Municipalities by Ingvild Røe, Maren Kristine Raknes Sogstad and Hilda Bø Lyng in Health Services Insights

sj-docx-3-his-10.1177_11786329251403887 – Supplemental material for Integrating Quality Improvement: A Qualitative Study of Leadership Approaches in Healthcare Services in Norwegian Municipalities Supplemental material, sj-docx-3-his-10.1177_11786329251403887 for Integrating Quality Improvement: A Qualitative Study of Leadership Approaches in Healthcare Services in Norwegian Municipalities by Ingvild Røe, Maren Kristine Raknes Sogstad and Hilda Bø Lyng in Health Services Insights

sj-pptx-4-his-10.1177_11786329251403887 – Supplemental material for Integrating Quality Improvement: A Qualitative Study of Leadership Approaches in Healthcare Services in Norwegian Municipalities Supplemental material, sj-pptx-4-his-10.1177_11786329251403887 for Integrating Quality Improvement: A Qualitative Study of Leadership Approaches in Healthcare Services in Norwegian Municipalities by Ingvild Røe, Maren Kristine Raknes Sogstad and Hilda Bø Lyng in Health Services Insights
