# Supplementary material for: Integrating Quality Improvement: A Qualitative Study of Leadership Approaches in Healthcare Services in Norwegian Municipalities
Source: Health Serv Insights. 2025 Dec 23;18:11786329251403887. doi: 10.1177/11786329251403887 (PMC12743786; doi:10.1177/11786329251403887)
Supplement: sj-doc-1-his-10.1177_11786329251403887 – Supplemental material for Integrating Quality Improvement: A Qualitative Study of Leadership Approaches in Healthcare Services in Norwegian Municipalities [file sj-doc-1-his-10.1177_11786329251403887.doc]

# Supplementary material: confirmatory letter. This is in Norwegian, English translation in parentheses)

# Vil du delta i forskningsprosjektet *Hvordan implementerer administrative ledere i kommunene kvalitetsforbedring?*

# (do you want to participate in the research project How are administrative leaders in municipalities implement quality improvement?)

# Dette er et spørsmål til deg om å delta i et forskningsprosjekt hvor formålet er å utforske nærmere hvordan administrative ledere på ulike nivå i kommunene arbeider for å implementere kvalitetsforbedring. I dette skrivet gir vi deg informasjon om målene for prosjektet og hva deltakelse vil innebære for deg.

# Formål (objectives)

Dette er et doktorgradsprosjekt hvor formålet er å utforske (explore) nærmere hvordan administrative ledere på ulike nivå i norske kommuner arbeider for å implementere kvalitetsforbedring. I prosjektet vil vi utforske følgende tema (themes):

# Hvordan jobber den enkelte leder konkret for å iverksette kvalitetsforbedring i sin kommune?

# Hva mener lederne er de største utfordringene med å få til/iverksette forbedringer? Hva gjør de for å møte utfordringene?

# Hvordan jobber lederne for å nå ut til i resten av organisasjonen? Hvordan sikrer de seg nødvendig forankring hos øvrige ledere og ansatte og hvordan forplikter de seg selv til arbeidet?

- Hvordan oppfatter ansatte lederes arbeid med kvalitetsforbedring?

**Hvem er ansvarlig for forskningsprosjektet?** (who is responsible for the research project?)

Senter for omsorgsforskning ved NTNU Gjøvik er ansvarlig for prosjektet. Doktorgradsstudien skal gjennomføres av Ingvild Røe, som er Offentlig sektor PhD og tilknyttet Senter for omsorgsforskning Gjøvik, og ansatt i c kommune*.*

**Hvorfor får du spørsmål om å delta? (**Why are you asked to participate?)

Du har blitt forespurt om å delta fordi du er ansatt som leder eller helsepersonell (leader or health worker) i kommunen og har derfor viktig kunnskap og erfaringer om hvordan ledere jobber med kvalitetsforbedring. Dine kontaktopplysninger er videreformidlet av NN, som er min kontaktperson i x kommune.

# Hva innebærer det for deg å delta? What does participating entail for you?

Prosjektet innebærer deltakelse i intervju og evt observasjon i møter (participate in interviews/observations) i kommunen der du kanskje deltar. Under har vi spesifisert hva deltakelse vil innebære for ulike grupper deltakere (specified for different groups):

*Til kommunedirektør, ansvarlig for helse og omsorgssektoren, enhetsledere og kvalitetsansvarlige:*

*(top leader, sector leader, unit managers and responsible for quality improvement):*

Hvis du velger å delta i prosjektet, innebærer det at du deltar i et intervju som tar ca. 45-60 min. Tema i intervjuet er dine erfaringer og vurderinger knyttet til kvalitetsforbedringsarbeidet i din enhet og helse- og omsorgstjenesten. Det vil bli utført lydopptak og tatt notater fra intervjuet.

*Til avdelingsledere og ansatte: (department managers and employees)*

Hvis du velger å delta i prosjektet, innebærer det at du deltar i et fokusgruppeintervju (ca 5 deltakere i gruppen), som varer opptil 90 min. Hensikten er å fange opp gruppens erfaringer og vurderinger knyttet til kvalitetsforbedringsarbeidet i din enhet. Det vil bli utført lydopptak og tatt notater fra intervjuet.

*Til alle vedr observasjon: (observation)*

I forbindelse med dette prosjektet ønsker jeg å delta som observatør i ledermøter eller samlinger hvor kvalitetsarbeid er et tema. Hensikten er å observere hvordan kvalitetsforbedring behandles og diskuteres i møtet/samlingen. Jeg tar notater fra møtet underveis. Hvilke møter dette gjelder, vil avtales på forhånd. Hvis du velger å delta i prosjektet, innebærer denne delen av prosjektet at du godtar at jeg deltar som observatør og tar notater fra møtet. Møtet vil ellers foregå på vanlig måte.

**Det er frivillig å delta** (participation is voluntary,

Det er frivillig å delta i prosjektet. Hvis du velger å delta, kan du når som helst trekke samtykket tilbake uten å oppgi noen grunn. Alle dine personopplysninger vil da bli slettet. Det vil ikke ha noen negative konsekvenser for deg hvis du ikke vil delta eller senere velger å trekke deg. (consent can be withdrawn at any time without consequence for you and information erased).

**Ditt personvern – hvordan vi oppbevarer og bruker dine opplysninger**

(your privacy – how we store and use your information)

Vi vil bare bruke opplysningene om deg til formålene vi har fortalt om i dette skrivet. Vi behandler opplysningene konfidensielt (confidential) og i samsvar med personvernregelverket (regulations on privacy). Intervjuene vil bli transkribert og videre bearbeiding og analyser av data fra intervju og observasjoner vil bli gjort ved hjelp av dataverktøyet NVIVO. Det vil være meg som student, mitt veilederteam og eventuell transkriberingsbistand som får tilgang til originale lydopptak og notater fra intervju og observasjoner

(access to original digital recordings and notes only for the researchers).

Navnet og kontaktopplysningene dine oppbevares separat fra datamaterialet. (name and data are stored separately). Når lydfilene og notatene er transkribert blir de slettet og datamaterialet avidentifiseres. Avidentifisert datamaterialet lagres på passord beskyttet området på NTNUs server. (After transcription the material is anonymized and kept on protected server by NTNU).

Det vil ikke være mulig å identifisere deg når resultatene fra studien publiseres. Prosjektansvarlig har ansvar for den daglige driften av forskningsprosjektet og at opplysninger om deg blir behandlet på en sikker måte.

(it will not be possible to identify you when the results are published).

**Hva skjer med personopplysningene dine når forskningsprosjektet avsluttes?**

(What happens with your information when the research project is over?)

Alle personopplysninger slettes ved prosjektslutt. Prosjektet vil avsluttes når PhD-oppgaven blir godkjent, estimert til høsten 2026. (All data will be destroyed, estimated to fall of 2026)

**Hva gir oss rett til å behandle personopplysninger om deg?**

**(**What gives us the right to treat information about you?)

Vi behandler opplysninger om deg basert på ditt samtykke (based on your consent). På oppdrag fra NTNU har Personverntjenester vurdert at behandlingen av personopplysninger i dette prosjektet er i samsvar med personvernregelverket. Corresponding with privacy regulations)

**Dine rettigheter** (your rights to see, correct, delete information when you can be identified)

Så lenge du kan identifiseres i datamaterialet, har du rett til:

- innsyn i hvilke opplysninger vi behandler om deg, og å få utlevert en kopi av opplysningene
- å få rettet opplysninger om deg som er feil eller misvisende
- å få slettet personopplysninger om deg
- å sende klage til Datatilsynet om behandlingen av dine personopplysninger

Hvis du har spørsmål til studien, eller ønsker å vite mer om eller benytte deg av dine rettigheter, ta kontakt med:

- Norges teknisk-naturvitenskapelige universitet, NTNU, fakultet for medisin og helsevitenskap (MH), Institutt for helsevitenskap Gjøvik ved Ingvild Røe, stipendiat, epost [inv@ringsaker.kommune.no](mailto:inv@ringsaker.kommune.no), tlf 93021551.
- NTNUs personvernombud: [Thomas Helgesen](https://www.ntnu.no/ansatte/thomas.helgesen), [thomas.helgesen@ntnu.no](mailto:thomas.helgesen@ntnu.no)

Hvis du har spørsmål knyttet til Personverntjenesters vurdering av prosjektet, kan du ta kontakt med:

- Personverntjenester på epost ([personverntjenester@sikt.no](mailto:personverntjenester@sikt.no)) eller på telefon: 53 21 15 00.

Med vennlig hilsen

Ingvild Røe

Stipendiat NTNU Senter for omsorgsforskning

/rådgiver Ringsaker kommune

Maren Sogstad,

Førsteamanuensis,

leder Senter for omsorgsforskning/

hovedveileder

**Samtykkeerklæring (consent form)**

# Jeg har mottatt og forstått informasjon om prosjektet *Hvordan implementerer administrative ledere i kommunene kvalitetsforbedring?* og har fått anledning til å stille spørsmål.

# (I have received and understood information about the project How administrative leaders in municipalities implement quality improvement).

# Jeg samtykker til/agree to

- å delta i intervju / fokusgruppeintervju (participate in interview)
- å delta i møter hvor det gjøres observasjoner (participate in meetings/observationss are conducted

Jeg samtykker til at mine opplysninger behandles frem til prosjektet er avsluttet

(I consent that my information is treated until the project is over)

Navn/enhet:

Name/unit

----------------------------------------------------------------------------------------------------------------

(Signert av prosjektdeltaker, dato) signature
