# Supplementary material for: Integrating Quality Improvement: A Qualitative Study of Leadership Approaches in Healthcare Services in Norwegian Municipalities
Source: Health Serv Insights. 2025 Dec 23;18:11786329251403887. doi: 10.1177/11786329251403887 (PMC12743786; doi:10.1177/11786329251403887)
Supplement: sj-docx-2-his-10.1177_11786329251403887 – Supplemental material for Integrating Quality Improvement: A Qualitative Study of Leadership Approaches in Healthcare Services in Norwegian Municipalities [file sj-docx-2-his-10.1177_11786329251403887.docx]

**Supplemental file**

**Interview and observation guide** (translated from Norwegian)

**Introduction to all informants:**

- About the project, the aim and main question (oral and written form).
- About the interview – to get the reflections and experiences, and what you do as a leader/employee, nice with examples
- The role of the interviewer is to listen and get an understanding, no right or wrong answer
- For focus group: specified purpose discussion between the informants, otherwise the same introduction as individual interviews.
- The interview is recorded, transcribed and kept safe according to the study protocol.
- The written consent form is filled out and signed before starting.

**Questions/themes for individual interviews:**

1. About you – title, responsibility, background, how long in this position
2. Tell me about how quality improvement goes on here? Organization, resources, participation, facilitation, plan/description/method. Current themes. Participate in networks or collaborate with others -about what? What is missing, and what is good?
3. How do you, as a leader/quality advisor, work to implement actions for QI in your unit? Role, follow up to ensure that it’s being done, apply any concrete tools?
4. Is QI vital/prioritized by you, among personnel, and the rest of the organisation? what do you do to increase/sustain this?
5. The regulation on leadership and QI, do you know this regulation? How do you apply this in your position? If not/in addition, other methods?
6. About the challenges which can affect how QI can be implemented: What do you think are the biggest challenges in your unit to accomplish improvement (enablers, barriers, for example, resources, organisation, frames, environment)? Do you do something to meet challenges? If so, what? If not, why?
7. Anchoring – how do you ensure anchoring in your unit, superior, subordinates - special actions?
8. Commitment by the single leader and worker to implement the improvement. What do you do to commit yourself – what contributes/limits the commitment? Your relationship to superiors/subordinates and their commitment
9. Personal motivation and workload (added questions about this after the first interviews)
10. Other themes not yet brought up?

Interviews were adapted somewhat to suit the participant's position/level.

**Observations in quality committees**

Introduction: my being there is cleared, will not cite from what they say, otherwise same introduction as interviews

1. Practicalities, date, participants and who led the meeting
2. About themes:
   1. What was discussed
   2. Was QI taken up
   3. Any reference to the regulation or other tools
   4. Talked about enablers or barriers
   5. Said anything about plans, routines
   6. Was responsibility or assignments given for following up
   7. Commitment and anchoring mentioned
   8. Other
3. General Atmosphere
4. Who talked – who not
5. Special themes discussed
6. Diversions or other themes than the agenda
