# Supplementary material for: Integrating Quality Improvement: A Qualitative Study of Leadership Approaches in Healthcare Services in Norwegian Municipalities
Source: Health Serv Insights. 2025 Dec 23;18:11786329251403887. doi: 10.1177/11786329251403887 (PMC12743786; doi:10.1177/11786329251403887)
Supplement: sj-pptx-4-his-10.1177_11786329251403887 – Supplemental material for Integrating Quality Improvement: A Qualitative Study of Leadership Approaches in Healthcare Services in Norwegian Municipalities [file sj-pptx-4-his-10.1177_11786329251403887.pptx]

## Slide 1
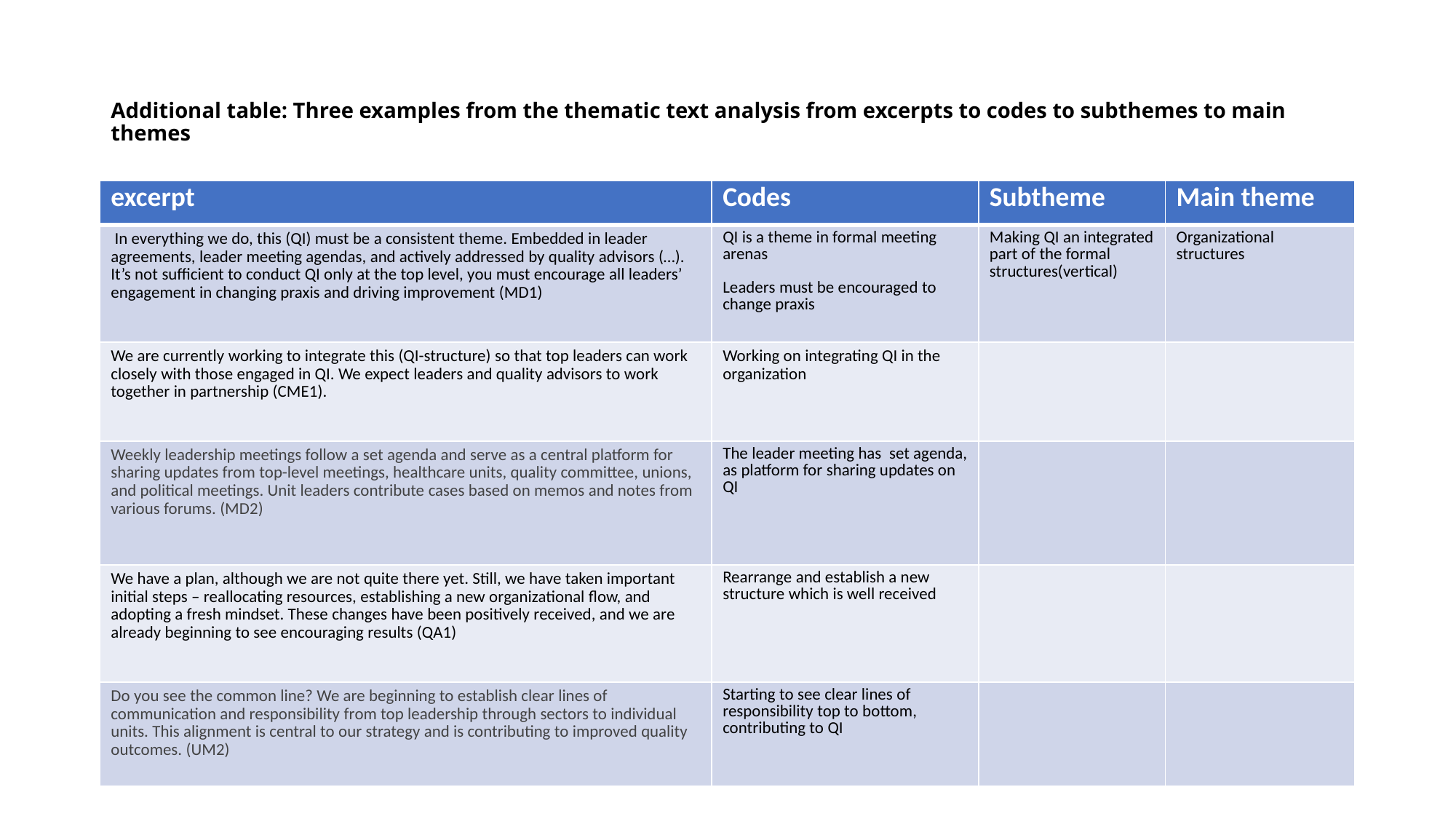

# Additional table: Three examples from the thematic text analysis from excerpts to codes to subthemes to main themes
| excerpt | Codes | Subtheme | Main theme |
| --- | --- | --- | --- |
| In everything we do, this (QI) must be a consistent theme. Embedded in leader agreements, leader meeting agendas, and actively addressed by quality advisors (…). It’s not sufficient to conduct QI only at the top level, you must encourage all leaders’ engagement in changing praxis and driving improvement (MD1) | QI is a theme in formal meeting arenas   Leaders must be encouraged to change praxis | Making QI an integrated part of the formal structures(vertical) | Organizational structures |
| We are currently working to integrate this (QI-structure) so that top leaders can work closely with those engaged in QI. We expect leaders and quality advisors to work together in partnership (CME1). | Working on integrating QI in the organization | | |
| Weekly leadership meetings follow a set agenda and serve as a central platform for sharing updates from top-level meetings, healthcare units, quality committee, unions, and political meetings. Unit leaders contribute cases based on memos and notes from various forums. (MD2) | The leader meeting has set agenda, as platform for sharing updates on QI | | |
| We have a plan, although we are not quite there yet. Still, we have taken important initial steps – reallocating resources, establishing a new organizational flow, and adopting a fresh mindset. These changes have been positively received, and we are already beginning to see encouraging results (QA1) | Rearrange and establish a new structure which is well received | | |
| Do you see the common line? We are beginning to establish clear lines of communication and responsibility from top leadership through sectors to individual units. This alignment is central to our strategy and is contributing to improved quality outcomes. (UM2) | Starting to see clear lines of responsibility top to bottom, contributing to QI | | |

## Slide 2
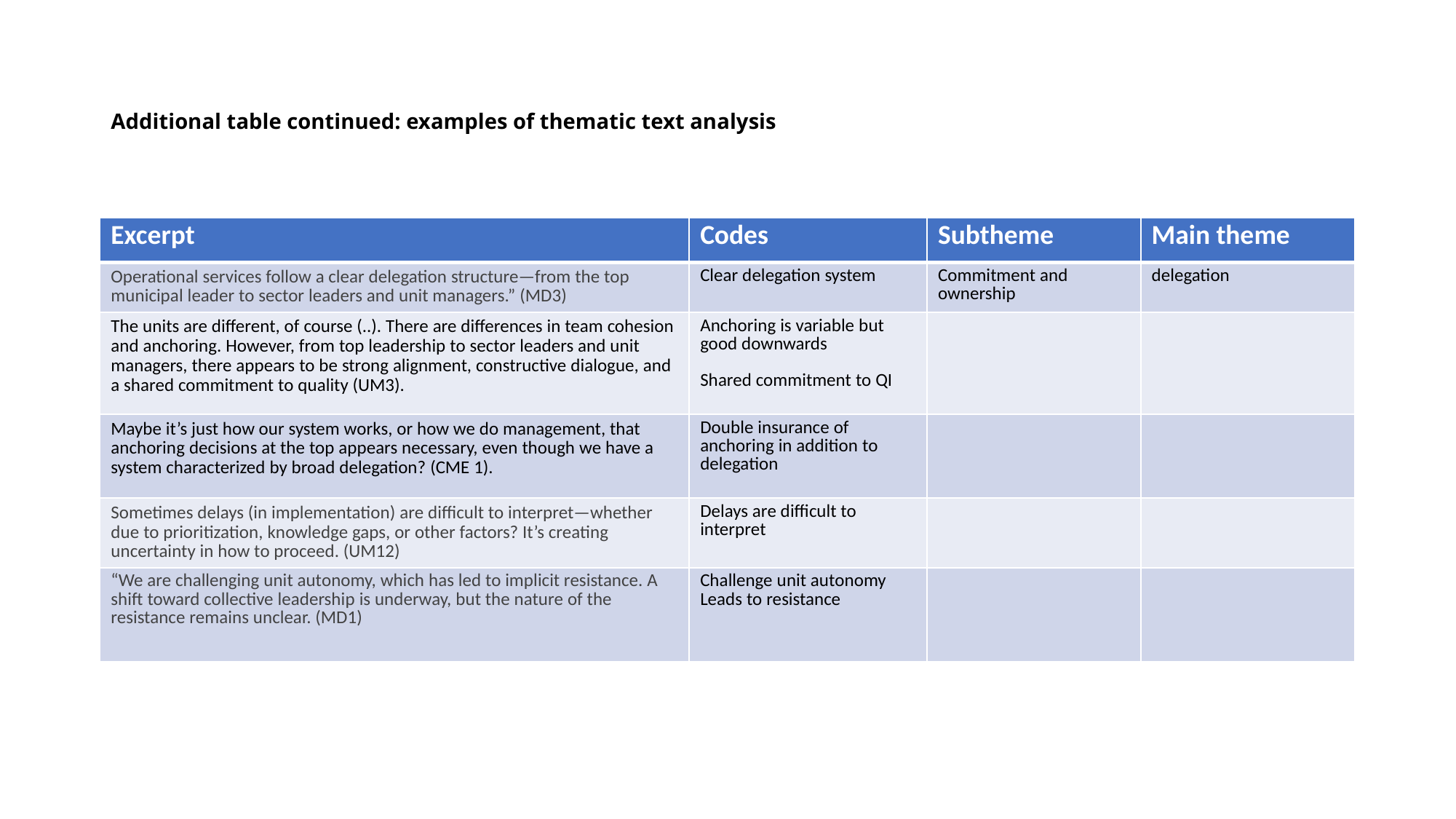

# Additional table continued: examples of thematic text analysis
| Excerpt | Codes | Subtheme | Main theme |
| --- | --- | --- | --- |
| Operational services follow a clear delegation structure—from the top municipal leader to sector leaders and unit managers.” (MD3) | Clear delegation system | Commitment and ownership | delegation |
| The units are different, of course (..). There are differences in team cohesion and anchoring. However, from top leadership to sector leaders and unit managers, there appears to be strong alignment, constructive dialogue, and a shared commitment to quality (UM3). | Anchoring is variable but good downwards Shared commitment to QI | | |
| Maybe it’s just how our system works, or how we do management, that anchoring decisions at the top appears necessary, even though we have a system characterized by broad delegation? (CME 1). | Double insurance of anchoring in addition to delegation | | |
| Sometimes delays (in implementation) are difficult to interpret—whether due to prioritization, knowledge gaps, or other factors? It’s creating uncertainty in how to proceed. (UM12) | Delays are difficult to interpret | | |
| “We are challenging unit autonomy, which has led to implicit resistance. A shift toward collective leadership is underway, but the nature of the resistance remains unclear. (MD1) | Challenge unit autonomy Leads to resistance | | |

## Slide 3
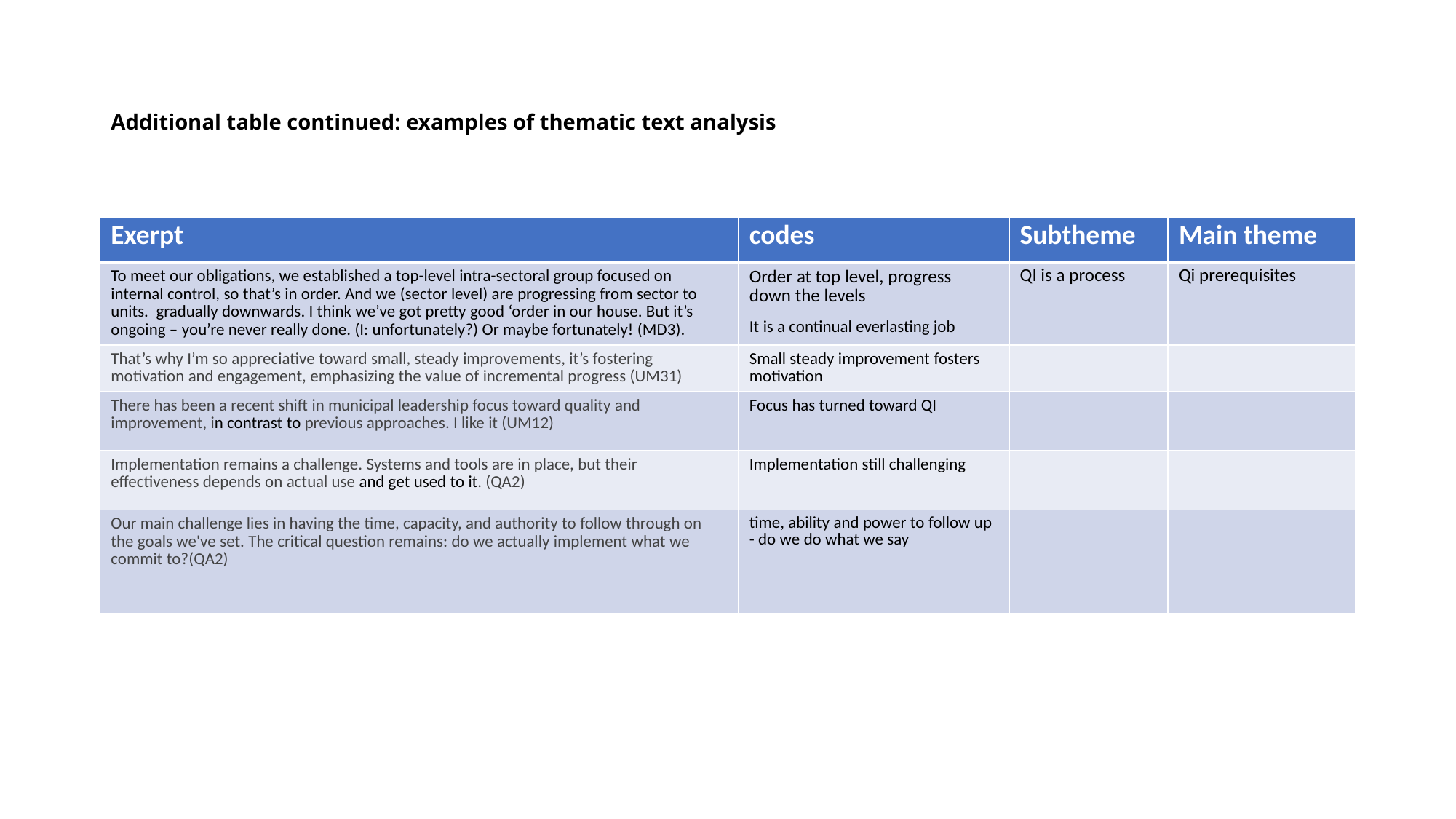

# Additional table continued: examples of thematic text analysis
| Exerpt | codes | Subtheme | Main theme |
| --- | --- | --- | --- |
| To meet our obligations, we established a top-level intra-sectoral group focused on internal control, so that’s in order. And we (sector level) are progressing from sector to units. gradually downwards. I think we’ve got pretty good ‘order in our house. But it’s ongoing – you’re never really done. (I: unfortunately?) Or maybe fortunately! (MD3). | Order at top level, progress down the levels It is a continual everlasting job | QI is a process | Qi prerequisites |
| That’s why I’m so appreciative toward small, steady improvements, it’s fostering motivation and engagement, emphasizing the value of incremental progress (UM31) | Small steady improvement fosters motivation | | |
| There has been a recent shift in municipal leadership focus toward quality and improvement, in contrast to previous approaches. I like it (UM12) | Focus has turned toward QI | | |
| Implementation remains a challenge. Systems and tools are in place, but their effectiveness depends on actual use and get used to it. (QA2) | Implementation still challenging | | |
| Our main challenge lies in having the time, capacity, and authority to follow through on the goals we've set. The critical question remains: do we actually implement what we commit to?(QA2) | time, ability and power to follow up - do we do what we say | | |
